# Effects of Multisensory Integration Training on Postural Stability Characteristics and Fall Risk in Older Adults: Systematic Review and Meta-Analysis

**DOI:** 10.2196/80345

**Published:** 2026-05-07

**Authors:** Chaoyu Guo, Lulu Yin, Peng Chen, Jianglong Zhan, Zhongqi Yu, Teck Cheng Tan, Yaru Wei, Yixue Gong, Menghan Xu, Van Minh Le, Lin Wang

**Affiliations:** 1 School of Exercise and Health, Shanghai University of Sport, Shanghai, China; 2 Sports Medicine and Rehabilitation Center, Shanghai University of Sport, Shanghai, China; 3 Shanghai Shangti Orthopaedic Hospital, 188 Hengren Road, Yangpu District, Shanghai, 200438, China, +86 13262592648

**Keywords:** multisensory integration training, older adults, postural stability, fall risk, meta-analysis

## Abstract

**Background:**

The risk of falls escalates with advancing age, a consequence of the concomitant degeneration of multiple physiological systems, altered sensory processing capabilities, and reduced postural control. Multisensory integration (MSI) training has been demonstrated to enhance the brain’s processing of multisensory information. However, existing studies show considerable variability in intervention duration and training modalities, limiting comparability across studies and contributing to inconsistent findings.

**Objective:**

This study aimed to systematically evaluate the effectiveness of MSI training on postural stability and fall risk in healthy older adults and provide an evidence base for clinical practice.

**Methods:**

Databases including PubMed, Embase, and Cochrane Library were searched by PRISMA (Preferred Reporting Items for Systematic Reviews and Meta-Analyses) guidelines. The methodological quality of the included randomized controlled trials was assessed using the Cochrane risk of bias tool, and publication bias was evaluated through funnel plots. Meta-analyses using R packages quantified effects via standardized mean differences (SMDs) and 95% CIs, with fixed or random effects models selected based on heterogeneity (*I*²). Subgroup analyses explored age, intervention duration, and type.

**Results:**

A total of 14 randomized controlled trials were included in the meta-analysis. Results showed that MSI training significantly reduced center of pressure displacement in the anterior-to-posterior displacement (SMD −1.64, 95% CI −2.78 to −0.49, *P*<.001) and center of pressure displacement in the medio-to-lateral displacement (SMD −1.37, 95% CI −2.68 to −0.07, *P*<.001). In terms of postural stability, MSI training significantly improved Berg Balance Scale scores (SMD 3.42, 95% CI 2.41 to 4.44, *P*=.006). In terms of fall risk, MSI training significantly reduced the time to complete the timed up and go test, and intervention type significantly moderated this effect (SMD −1.43, 95% CI −2.36 to −0.50, *P*<.001). Additionally, MSI training reduced the risk of falls (SMD −1.27, 95% CI −2.03 to −0.52, *P*<.001).

**Conclusions:**

In conclusion, MSI training is an effective intervention for enhancing static and dynamic postural control and reducing fall risk in healthy older adults, suggesting a beneficial effect on postural stability and fall-related outcomes.

## Introduction

According to the World Health Organization, the risk of falls among older adults increases significantly with age [1], and falls impose substantial societal burdens, both directly and indirectly, including increased demands on family caregiving and health care resources [2]. These events are driven by a combination of age-related physiological decline and underlying pathological factors. It is worth noting that aging is not a single-system degeneration but a complex process of cognitive, musculoskeletal, sensory processing, and other multisystems synergistic decline [3]. On the one hand, sensory-motor system function decreases with aging; on the other hand, aging also leads to changes in the way of processing sensory information in the central nervous system [4-6]. The cognitive demands required to maintain balance during challenging postural tasks increase in older adults [7-9]. In addition, decreased brain volume and structural changes in the brain, such as gray matter atrophy and decreased white matter integrity, affect the transmission and integration of sensory information in older adults [10,11]. At the same time, changes in neurotransmitter levels interfere with sensory information transmission and processing [12,13]. In particular, functional and connectivity declines in core multisensory brain regions, such as parietal and temporal cortices, reduce multisensory integration in older adults, which in turn affects postural stability and fall risk [8,14,15]. Deficits in postural control are the most important risk factor for falls in older adults, directly weakening the physiological basis for maintaining postural stability and significantly increasing fall risk. Understanding the effects of multisensory integration (MSI) training on postural control is essential to address fall risk and improve quality of life in older adults.

The maintenance of postural stability relies on the dynamic coordination of multisensory integration, in which the brain integrates and assigns weights to visual, vestibular, and somatosensory information in real time [16]. It is important to note that the decline in multisensory integration is not irreversible [17,18], and MSI training is a method to optimize the brain’s ability to process multisensory information through targeted interventions. This type of training usually combines visual, vestibular, and somatosensory stimuli to design a variety of tasks [19-21]. Through repeated practice, the nervous system can readjust the weights of different sensory inputs and strengthen the efficiency of integrating sensory information, thus improving postural control and balance stability [22,23]. Studies have shown that MSI training can be beneficial in improving balance function in older adults and can restore sensory integration deficits in patients with neurological impairments [22,23]. Allison et al [24] found that MSI training significantly improved postural stability and fall risk in older adults; however, Dowd et al [25] did not find that MSI training could improve postural stability and fall risk in older adults. Existing studies have found that the length and type of interventions in training programs vary greatly, resulting in limited comparability of results, and their methodological differences may affect the consistency of validation of intervention effects [26-28].

Drawing on previous literature [29-33], when analyzing the effects of interventions targeting memory impairment or balance in older adults, researchers commonly use comparisons based on age stratification, intervention type classification, and intervention duration staging. Combined with regression analysis to examine the influence of these variables on effect sizes, this methodological framework provides a reliable reference for interpreting the heterogeneity observed in this study. Therefore, this study aimed to integrate the existing evidence to quantify the effects of MSI training on postural stability and fall risk in older adults through systematic evaluation and meta-analysis, and to reveal the sources of heterogeneity through subgroup analyses of age, duration of intervention, and intervention type, so as to provide evidence-based medical evidence for the application of MSI training in older adults.

## Methods

This systematic review and meta-analysis was conducted in accordance with the PRISMA (Preferred Reporting Items for Systematic Reviews and Meta-Analyses) checklist and registered on PROSPERO on April 1, 2025 (CRD420251023158).

### Search Strategy

Two authors (CG and YW) individually conducted a literature retrieval in 5 electronic databases—PubMed, Embase, Cochrane Library, WoS, and Scopus—from the inception of each database up to April 5, 2025, without language restriction. Medical Subject Headings (MeSH) terms or keywords were used during the literature search. The search terms were (“sensory reweight*” OR “sensory recalibrat*” OR “multisensory integrat*” OR “sensory train*“) AND (“elderly*” OR “older adult*“) AND (“balance” OR “falls” OR “fall risk”), and the detailed search strategy is shown in Multimedia Appendix 1. After the search was completed, all search results were imported into EndNote reference management software (Clarivate) for deduplication, and the reference lists of full-text literature were manually searched to identify studies that were not retrieved through the database search.

### Research Inclusion and Exclusion Criteria

The study eligibility criteria are shown in Textbox 1.

Textbox 1. **Study eligibility criteria.**

**Inclusion criteria**

- Participants: healthy older adults aged ≥60 years, with no musculoskeletal dysfunction within the past 6 months, no confirmed peripheral somatosensory or vestibular deficits, visual acuity sufficient for daily activities, and a Montreal Cognitive Assessment score ≥26 or a Mini-Mental State Examination score ≥24
- Interventions: the intervention was multisensory integration (MSI) training, defined as any protocol that simultaneously manipulates or stimulates ≥2 sensory modalities (visual, vestibular, and somatosensory) to provoke central sensory integration through conflict or reweighting
- Control group: presence of a healthy control group to establish baseline changes and accurately identify the effects of MSI training
- Outcome measures: the primary outcome was center of pressure (COP) displacement (including anterior-to-posterior and medio-to-lateral displacements). Secondary outcomes included (1) postural stability measures under different test conditions (single-leg standing, 2-legged tandem standing, and 2-legged standing), specifically COP displacement and Berg Balance Scale scores; and (2) fall risk prediction indicators, including direct risk measures (fall risk, times/year) and functional measures (timed up and go test), and total intervention duration (weeks)

**Exclusion criteria**

- Nonrandomized controlled trials
- Insufficient data for analysis
- Duplicate publications
- Non–peer-reviewed literature
- Serious methodological flaws, defined as a lack of adequate randomization or allocation concealment, intergroup cross-over contamination, use of invalid or noncomparable outcome measures, or dropout rates exceeding 20% without intention-to-treat analysis

The summary of outcome measures is shown in Table 1.

**Table 1.** Summary of outcome indicators.

Specific tests		Assessment of indicators
Postural stability test		
	Single-leg standing test	AP^a^ displacement^b^, ML^c^ displacement^d^, and BBS^e^ score
	2-legged tandem standing test	AP displacement, ML displacement, and BBS score
	2-legged standing test	AP displacement, ML displacement, and BBS score
Fall risk prediction		
	Functional test metrics	TUG^f^ time
	Risk prediction indicators	Fall risk (time/year)

a AP: anterior-to-posterior.

b Center of pressure displacement in the anterior-to-posterior.

c ML: medio-to-lateral.

d Center of pressure displacement in the medio-to-lateral.

e BBS: Berg Balance Scale.

f TUG: timed up and go.

Two reviewers independently screened the titles, abstracts, and full texts of the identified studies. Any disagreements were resolved through consultation with a third adjudicator, who made the final determination based on the predefined criteria. Relevant data, including study design, population characteristics, sample size, intervention details, and outcome measures, were extracted into a standardized Microsoft Excel 2024 (Microsoft Corporation) form.

### Quality and Risk of Bias Assessment

Quality assessment was performed using the Cochrane risk-of-bias tool (Cochrane), which consists of seven domains: (1) random sequence generation, (2) allocation concealment, (3) blinding of experimental staff, (4) blinding of results staff, (5) data integrity, (6) selective reporting of results, and (7) other sources of bias. For each source of bias, studies were classified as low, high, or unclear (if reporting is insufficient to assess a specific domain) risk. Two independent reviewers (CG and YW) assessed the results. In case of disagreement, differences of opinion were discussed and resolved with a third researcher (MX).

### Data Extraction

Literature screening and data extraction were carried out using an independent double-blind method. Two researchers, CG and YW, first screened the literature according to the titles and abstracts of the literature, excluded obviously irrelevant studies, then read the full text of the potentially relevant literature, and determined the final selection of the literature according to the inclusion and exclusion criteria. In case of disputes during the screening process, a third researcher (MX) was involved in the discussion and decided. Data extracted included the first author, year of publication, sample size, age of subjects, intervention plan and specific protocol, outcome measures, and major findings. If the original text did not contain the target data or the information was ambiguous, an email was sent to contact the corresponding author for assistance or confirmation.

### Statistical Analysis

Meta-analyses were performed using the meta and metafor packages in the statistical software R (version 4.2.0; R Foundation for Statistical Computing). The effect size for continuous outcomes was expressed as the standardized mean difference (SMD), specifically Hedges g to correct for small-sample bias. Effect magnitudes were interpreted using Cohen conventions (0.2=small, 0.5=medium, and ≥0.8=large). This study adopted “95% CI not including zero” as the criterion for determining statistically significant differences between groups. The heterogeneity between studies was assessed by the *I²* statistic. When *I²*≤50%, the heterogeneity between studies was considered low, and meta-analysis was conducted using the fixed-effects model; when *I*²>50%, the heterogeneity between studies was considered high, and meta-analysis was conducted using the random-effects model. Age stratification was based on the age distribution of the study population, dividing participants into 60 to 70 years and ≥70 years groups [34]. Intervention duration was categorized according to the median study period and the stabilization phase of multisensory training effects [35]. Intervention types were classified based on the core modalities (dynamic, static, or combined dynamic-static) used in the included studies, combined with clinical classification standards for balance training. Additionally, regression analysis was conducted to explore key findings of this study. Meta-regression models were fitted within a random-effects framework, and the significance of regression coefficients for each variable was tested to determine whether statistical differences existed in effect sizes between different intervention types. Sensitivity analyses were conducted by excluding the included studies one by one to assess the effect of individual studies on the overall results and to judge the reliability of the results. Funnel plots were used to assess publication bias, and the results were interpreted with caution if publication bias existed, and further unpublished studies were searched or statistically corrected if necessary.

## Results

### Study Selection and Characteristics

**Figure 1.**
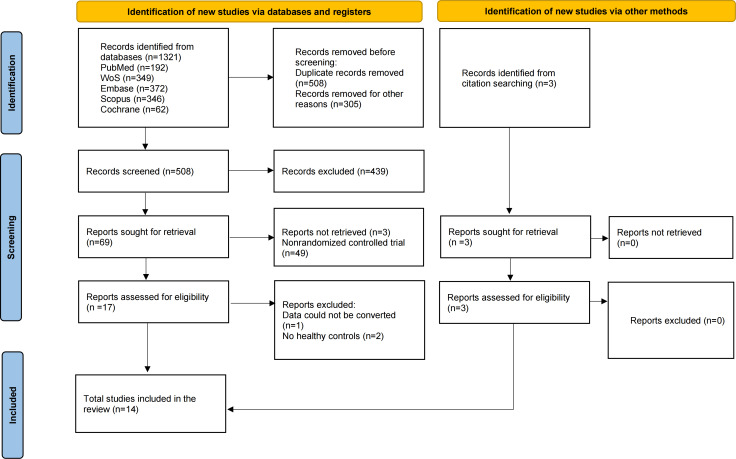
PRISMA (Preferred Reporting Items for Systematic Reviews and Meta-Analyses) flow diagram of the study selection process.

A total of 1321 records were obtained through systematic search of multiple databases. After deleting duplicates, screening titles and abstracts, and reading the full text, 14 studies were finally included. The specific screening process is shown in Figure 1.

**Figure 2.**
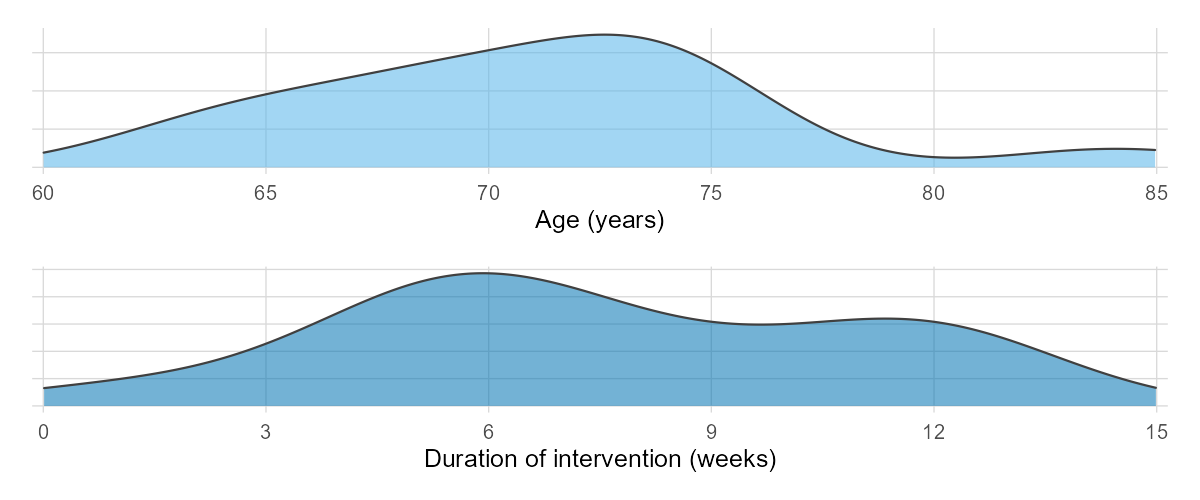
Distribution maps of age and intervention duration.

Included studies were published from 2010 to 2025, with a total sample size of 1321 cases, and the type of study was randomized controlled trial. The outcome metrics covered anterior-to-posterior (AP) displacement (5 items) [11,35-38], medio-to-lateral (ML) displacement (4 items) [11,35,36,38], BBS score (3 items) [39-41], TUG (7 items) [35,39,41-45], fall risk (4 items) [37,45-47], and the basic characteristics of each study are shown in Multimedia Appendix 1. Among the included studies, the age of the experimental group was mainly concentrated in 2 intervals, 60 to 70 years and >70 years, and the intervention time was mainly distributed in the period of <6 weeks and >6 weeks. The types of interventions were mainly dynamic balance training, static balance training, and combined dynamic and static balance training, and the distribution is shown in Figure 2.

**Figure 3.**
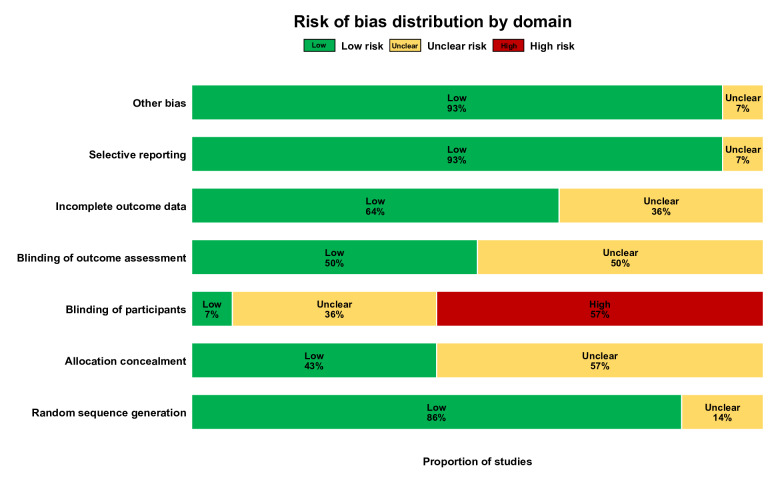
Distribution of risk of bias across assessment domains.

Of the 14 included studies, 12 [35,36,38-47] described specific methods of random sequence generation, 6 [11,36,37,40,42,47] described allocation concealment, and 7 [35,37,39,42,44,46,47] were single-blind studies. However, 9 studies [11,35,38-41,43,45,46] reported detachment without specifying the reason, as shown in Figures3  4.

**Figure 4.**
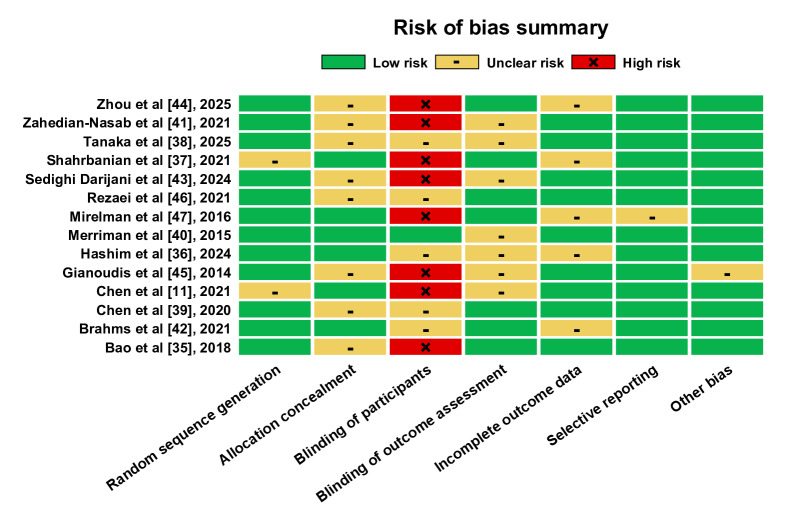
Results of the risk of bias assessment of the included studies.

### Main Results

**Figure 5.**
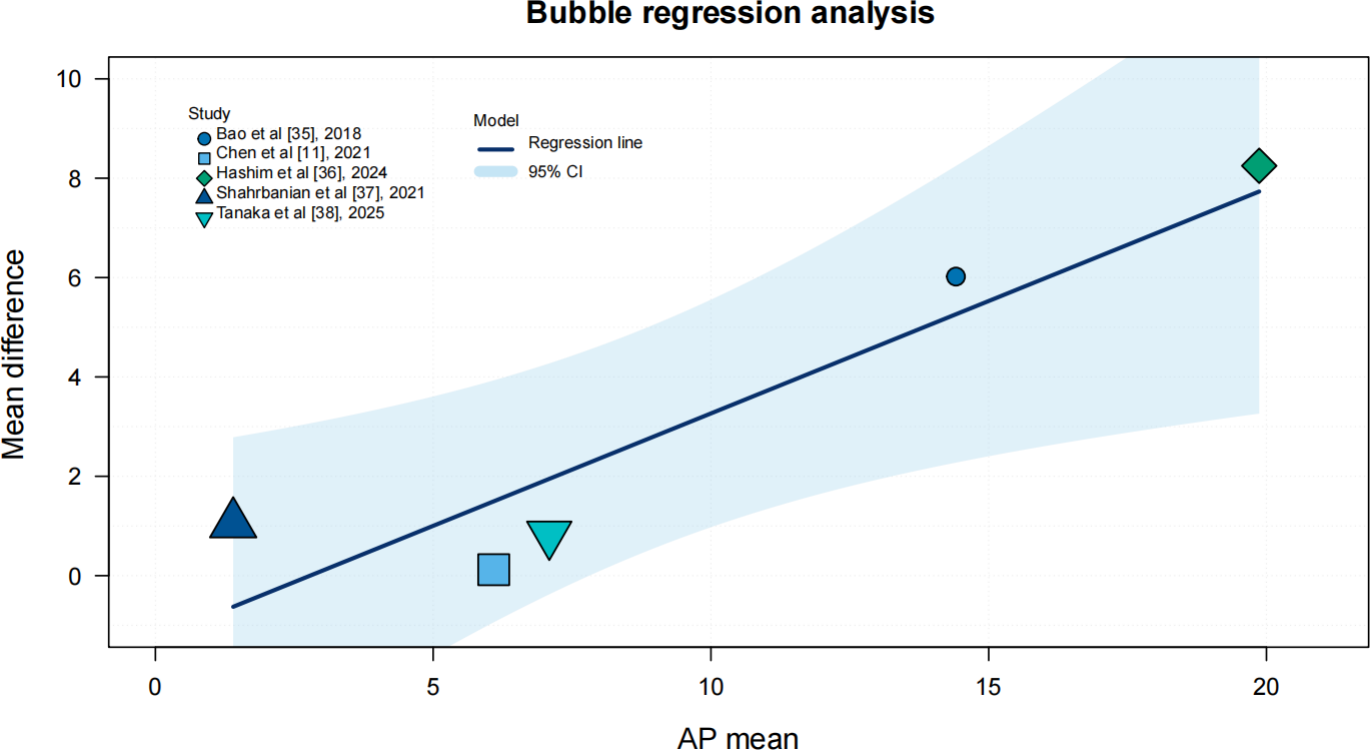
Bubble chart of the regression analysis of anterior-to-posterior (AP) displacement based on Bao et al [35], Chen et al [11], Hashim et al [36], Shahrbanian et al [37], and Tanaka et al [38].

**Figure 6.**
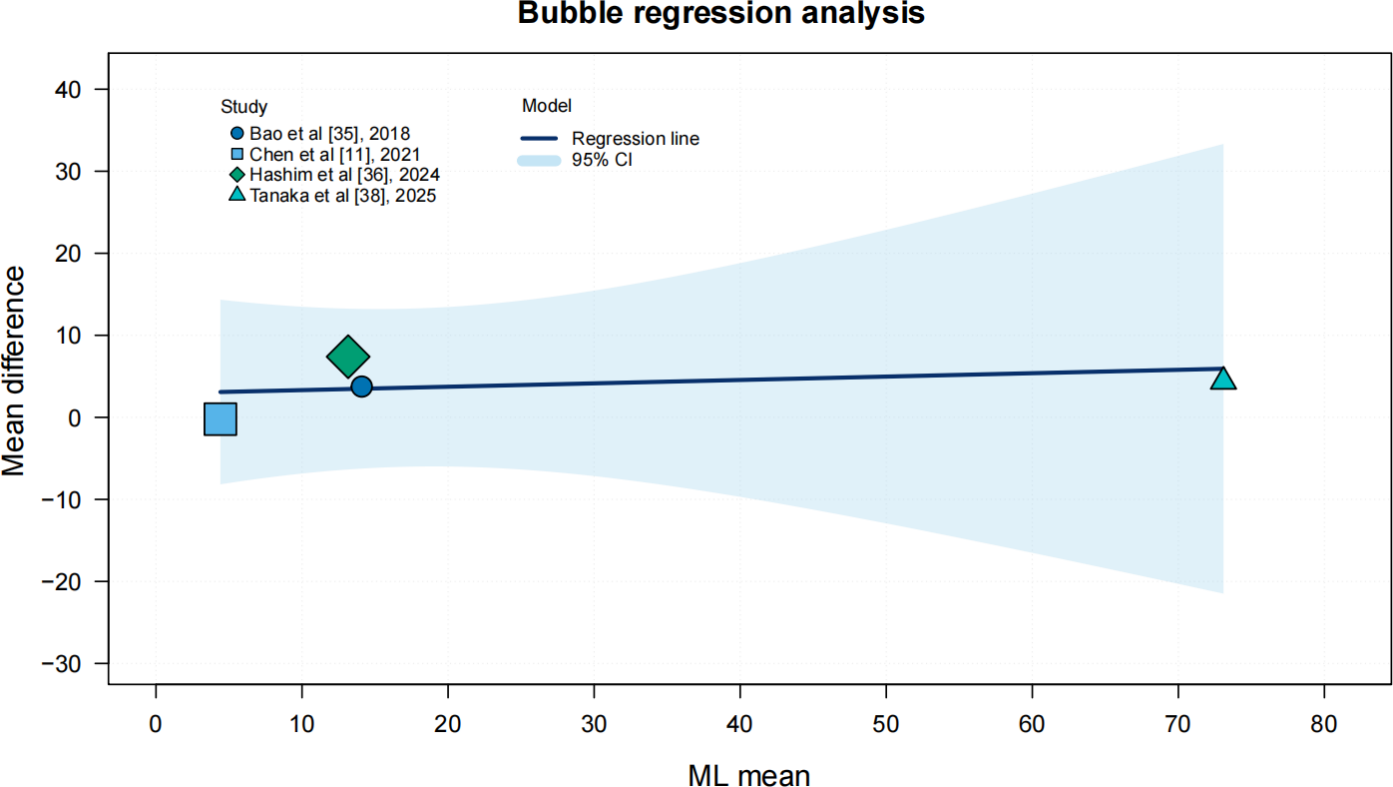
Bubble chart of the regression analysis of medio-to-lateral (ML) displacement based on Bao et al [35], Chen et al [11], Hashim et al [36], and Tanaka et al [38].

MSI training significantly improved AP displacement (SMD −1.64, 95% CI −2.78 to −0.49; *I²*=83%, *P*<.001), and ML displacement showed a significant reduction (SMD −1.37, 95% CI −2.68 to −0.07; *I²*=83%, *P*<.001). In subgroup analyses, age and intervention duration showed no significant moderating effects on AP or ML displacement. In contrast, the type of intervention significantly moderates AP displacement (*P*=.009) but not ML displacement. Additionally, we conducted regression analyses using study-level data points to validate these findings and to illustrate the observed distribution of effects across studies. Sensitivity analysis by excluding studies one by one revealed that the pooled effect size of AP displacement fluctuated between −3.41 and −1.45 and that of ML displacement fluctuated between −3.83 and −0.4. All effect sizes after exclusion iterations remained statistically significant (95% CIs did not include 0), indicating that the main results were robust, as shown in Figure 5, Figure 6, Multimedia Appendix 1, and Table 2.

**Table 2.** Summary of the results of the included studies.

Outcomes	Studies		*I*^2^ statistic, %	Subgroup, *P* value			Sensitivity analysis
	Reference (n)	SMD^a^ (95% CI)		Age (years)	Duration of intervention	Type of intervention	
AP^b^ displacement^c^	5 [11,35-38]	−1.64 (−2.78 to −0.49)	83	.13	.09	.009	Stable
ML^d^ displacement^e^	4 [11,35,36,38]	−1.37 (−2.68 to −0.07)	83	.09	.58	.09	Stable
TUG^f^ time	7 [35,39,41-45]	−1.43 (−2.36 to −0.50)	89	.26	.24	*<.001^g^*	Stable
BBS^h^ score	3 [39-41]	3.42 (2.41 to 4.44)	0	N/A^i^	N/A	N/A	N/A
Fall risk	4 [37,45-47]	−1.27 (−2.03 to −0.52)	99	.07	.06	.001	Stable

a SMD: standardized mean difference.

b AP: anterior-to-posterior.

c Center of pressure displacement in the anterior-to-posterior.

d ML: medio-to-lateral.

e Center of pressure displacement in the medio-to-lateral.

f TUG: timed up and go.

g Statistically significant.

h BBS, Berg Balance Scale.

i N/A: no subgroup or sensitivity analysis was carried out due to insufficient data.

### Secondary Results

#### Effect of MSI Training on Postural Stability in Older Adults

There was low heterogeneity in the studies of BBS scores (SMD 3.42, 95% CI 2.41 to 4.44; *I²*=0%, *P=*.006), suggesting that the MSI training significantly improved BBS scores of the older adults and had reliable effects on the comprehensive quantitative static and dynamic balance enhancement, with a high degree of consistency among different studies. The results of the sensitivity analyses were not stable, and the funnel plot did not show obvious symmetry, suggesting that the results of the studies were influenced by individual studies, as shown in Multimedia Appendix 1 and Table 2.

#### Effect of MSI Training on Fall Risk in Older Adults

High heterogeneity was present in the studies of time to TUG completion (SMD −1.43, 95% CI −2.36 to −0.50; *I*²=89%, *P*<.001). Subgroup analysis by intervention type was statistically significant (*P*<.001), and sensitivity analysis results were stable. The funnel plot analysis showed good symmetry, suggesting that the results were relatively reliable. Fall risk–related studies had extremely high heterogeneity (SMD −1.27, 95% CI −2.03 to −0.52; *I²*=99%, *P*<.001). Subgroup analyses by intervention type was significant (P=.001), whereas age and intervention duration were not significant and the results of sensitivity analyses were stable, but the funnel plot showed asymmetry, as shown in Multimedia Appendix 1 and Table 2.

## Discussion

This systematic review integrated data from 14 randomized controlled trials and demonstrated that MSI training significantly improved postural stability and reduced fall risk in older adults. Specifically, MSI training led to reductions in AP and ML displacement. However, high heterogeneity was observed in certain outcomes, which may be attributed to methodological variations, participant diversity, and differences in intervention protocols.

### Effect of MSI Training on Postural Stability

Although MSI training significantly improved both AP and ML displacements, the high heterogeneity limits the generalizability of these findings. In terms of the intervention protocols, the training methods used in different studies varied greatly. Bao et al [35] used vibro-tactile sensory enhancement interventions, whereas Chen et al [11] used stroboscopic training in balance training; however, meta-regression and subgroup analyses did not identify significant moderating effects of age, intervention duration, or training type on these outcomes. For AP displacement, intervention type showed a significant moderating effect, whereas age and intervention duration did not. For ML displacement, no significant moderating effects were observed, suggesting that other moderating factors (eg, baseline balance ability or cognitive status) may be at play [13,22]. Future studies should standardize intervention protocols to better elucidate the mechanisms by which different training modalities influence balance performance in older adults.

Notably, BBS scores showed consistent improvement with low heterogeneity, indicating that MSI training effectively enhances overall balance control. This aligns with the concept that MSI training promotes central sensory recalibration and optimizes sensorimotor integration. This was attributed to the enhancement of the integration efficiency of the central nervous system by training, which optimized the distribution of sensory weights and thus improved postural control strategies. In a study by Eikema et al [48], it was found that older adults had a more pronounced decline in central sensory integration and a longer effective adaptive world than younger adults when faced with a challenging task. This finding provides an important reference for the development of stratified and individualized interventions in clinical practice based on baseline sensory function and age characteristics of older adults.

### Effect of MSI Training on Fall Risk

There is a moderate heterogeneity in the study of the time of completion of the TUG test, and the results of the analysis of the subgroup of the intervention type are significant, which suggests that there is a significant difference in the effects of the different types of interventions on the ability of the older adults to rise and walk, which is clinically important for assessing the effect of training on functional mobility improvement. The results of the subgroup analyses were significant, indicating that the effects of different intervention types on the ability to get up and walk differed significantly, which is clinically important for evaluating the effects of training on the functional mobility of older adults. In addition, the overall heterogeneity of the studies on fall risk was extremely high, and most of the studies had small sample sizes, which resulted in insufficient statistical validity and limited the reliability and extrapolation of the results.

### Mechanism of Action of MSI Training

With advancing age, balance and postural control become increasingly vision dependent, as neurodegenerative processes impair vestibular and somatosensory function. Additionally, concomitant neuronal loss and pathway damage further hinder afferent signaling [8,49,50]. MSI training is designed to mitigate these age-related deficits by jointly targeting peripheral sensory function, central integrative efficiency, and neuromuscular coordination [4,51-53]. At the peripheral level, structured visual exercises, such as object recognition under varied illumination and complex backgrounds, have been reported to improve visual acuity and the perception of shape, displacement, and motion [51,52]. Graded vestibular stimuli, including slow head rotations on stable surfaces and gentle tilting or rotation using dedicated apparatus, enhance responsiveness to head and trunk movements [54,55], whereas somatosensory tasks requiring precise limb positioning and force regulation strengthen proprioceptive feedback and movement accuracy [4,53]. These peripheral adaptations are accompanied by central reweighting of multimodal inputs: exposure to diverse sensory environments fosters more efficient integration of visual, vestibular, and somatosensory information [16,51], thereby accelerating postural adjustments and improving stability during dynamic tasks such as walking in simulated complex settings [56-58].

Concurrently, MSI training refines neuromuscular coordination by tightening the coupling between neural commands and muscular execution, manifested as smoother signal transmission, more accurate and rapid muscle responses, and reduced activation latency [56,59-61]. Bugnariu et al [62] reported that older adults exhibited 40 to 60 ms longer electromyographic latencies in the distal-to-proximal activation sequence compared to younger adults. Notably, a single 1-hour MSI session reduced ankle muscle latency by approximately 20 ms, suggesting improved neuromuscular timing [63]. Beyond cortical contributions, MSI training engages the cerebellum, basal ganglia, and other motor control centers: cerebellar metabolic activity increases during training, facilitating consolidation of motor skills, while the basal ganglia modulate initiation and termination of motor programs to enhance movement smoothness and precision [64]. Consistent with this systems-level account, Wang et al [17] reported less adaptive brain connectivity in older vs younger adults and demonstrated that MSI training can augment functional connectivity and neural adaptability, supporting structural and functional central adaptation and establishing a robust neuromodulator network for motor improvement [65]. Taken together, by strengthening sensory inputs, optimizing central integration, and improving neuromuscular coordination, MSI training constitutes a comprehensive and effective approach to enhancing postural stability in older adults, thereby reducing fall risk and improving quality of life [37].

### Clinical Significance and Future Prospects of the Study

This study shows that MSI training is important for improving postural stability and reducing fall risk in older adults and provides a theoretical basis and practical guidance for clinical rehabilitation and geriatric care. To enhance the reliability and practicality of the results, future studies should expand the sample size and conduct multicenter studies to include older adults with different health conditions to verify the generalizability of the effect and continue to follow-up and evaluate various indicators to investigate the long-term effect, the best training mode, and the applicability to older adults with different health conditions, and the mechanism of action can be analyzed by combining neuroimaging and muscle electrophysiology techniques. We will continue to follow-up and evaluate various indicators to investigate the long-term effect, the optimal mode, and the applicability to different health conditions of older adults, to provide more accurate and effective interventions.

### Comparison With Other Studies

Previous studies [22,66] have confirmed that although single balance or strength training can improve static postural stability, the lack of multisensory conflict simulation makes it difficult to transfer motor adaptations to dynamic multitasking scenarios and fails to effectively reduce the risk of falls. This issue was broken through in 2 systematic review studies [27,28] that used qualitative research to propose that MSI training significantly improves postural control, a finding that is highly compatible with the results of this study.

This study realizes dual innovations at the methodological and outcome levels. In terms of methodology, we broke through the limitations of previous qualitative studies and adopted a combination of qualitative and quantitative analysis to construct a more rigorous research system by integrating multidimensional data. In contrast to the study by Sápi et al [67], which concluded that MSI training could not improve the COP displacement in the AP direction in older adults, this study, by incorporating the dynamic balance task and revamping the assessment scenarios, confirms that MSI training has a significant improvement in the COP displacement in the AP and ML directions, which is more in line with the real functional needs of older adults.

The innovative value of this study lies in the fact that it is the first quantitative study to systematically demonstrate the effectiveness of MSI training on the optimization of omnidirectional postural control and the reduction of fall risk, which not only provides a quantitative theoretical basis for the prevention of falls in older adults but also has a guiding significance for clinical practice, suggesting that individual functional shortcomings need to be based on individual design of differentiated MSI training programs integrating static and dynamic tasks, so as to accurately improve the motor function and quality of life of older adults. This finding not only provides a quantitative theoretical basis for fall prevention but also guides clinical practice.

### Study Quality and Limitations

Several limitations should be considered when interpreting our findings. First, evidence on long-term effectiveness remains limited, as only 3 trials provided follow-up data, and their high heterogeneity precluded meta-analysis. Second, significant methodological and clinical heterogeneity, including variations in randomization, participant characteristics, intervention protocols, and outcome measures, may affect comparability across studies, though we addressed this through random-effects models and subgroup analyses. Third, most trials had small sample sizes, which could overestimate effect sizes and reduce statistical power; sensitivity analyses were performed to ensure robustness. Finally, potential publication bias, as indicated by funnel plot asymmetry, cannot be ruled out despite comprehensive search efforts. Future studies should adopt standardized interventions and assessments, include longer follow-up periods, and explore moderating effects of individual characteristics to strengthen the evidence base for clinical practice.

### Conclusion

MSI training can improve postural stability and reduce fall risk in older adults, but we need to conduct long-term follow-up of multicenter and large-sample studies to identify the best intervention mode. Meanwhile, standardization of measurement tools, optimization of intervention protocols, attention to individual differences, and evaluation of long-term effects will help to improve the comparability of studies and provide more accurate guidance for clinical practice.

## Supplementary material

10.2196/80345 Multimedia Appendix 1 Supplementary materials, including search strategies, characteristics of included studies, additional analyses, figures, statistical code, dataset, and references.

10.2196/80345 Checklist 1 PRISMA checklist.

## Abbreviations

AP: anterior-to-posterior
MeSH: Medical Subject Headings
ML: medio-to-lateral
MSI: multisensory integration training
TUG: timed up and go
