# Supplementary material for: Effects of Multisensory Integration Training on Postural Stability Characteristics and Fall Risk in Older Adults: Systematic Review and Meta-Analysis
Source: JMIR Aging. 2026 May 7;9:e80345. doi: 10.2196/80345 (PMC13152038; doi:10.2196/80345)
Supplement: Multimedia Appendix 1 [file aging-v9-e80345-s001.docx]

**Supplementary materials**

[**Appendix A. Search strategies** 1](#_Toc228475386)

[**PubMed** 1](#_Toc228475387)

[**Cochrane Library** 1](#_Toc228475388)

[**EMBASE** 1](#_Toc228475389)

[**Web of Science** 2](#_Toc228475390)

[**Scopus** 2](#_Toc228475391)

[**Appendix B. Characteristics of included studies** 3](#_Toc228475392)

[**Appendix C. Forest plot summary** 9](#_Toc228475393)

[**Appendix D. Meta-regression** 14](#_Toc228475394)

[**Appendix F. Research dataset** 18](#_Toc228475395)

[**References cited in Multimedia Appendix 1** 20](#_Toc228475396)

**Appendix A. Search strategies**

**PubMed**

| **Concept** | **Query** | **Hits** |
| --- | --- | --- |
| #1 | ("sensory reweighting"[Title/Abstract] OR "sensory reweight*"[Title/Abstract] OR "sensory recalibration"[Title/Abstract] OR "sensory recalibrat*"[Title/Abstract] OR "multisensory integration"[Title/Abstract] OR "multisensory integrat*"[Title/Abstract] OR "multisensory combination"[Title/Abstract] OR "multisensory combinat*"[Title/Abstract] OR "sensory fusion"[Title/Abstract] OR "sensory fus*"[Title/Abstract] OR "sensory training"[Title/Abstract] OR "sensory train*"[Title/Abstract] OR "sensory strategy"[Title/Abstract] OR "sensory strateg*"[Title/Abstract] OR "sensory processing"[Title/Abstract] OR "sensory process*"[Title/Abstract] OR "sensory adaptation"[Title/Abstract] OR "sensory adapt*"[Title/Abstract]) AND ("aging"[Title/Abstract] OR "age*"[Title/Abstract] OR "elderly"[Title/Abstract] OR "elderly*"[Title/Abstract] OR "older adult"[Title/Abstract] OR "older adult*"[Title/Abstract] OR "senior"[Title/Abstract] OR "senior*"[Title/Abstract] OR "geriatrics"[Title/Abstract] OR "geriatr*"[Title/Abstract] OR "aged population"[Title/Abstract] OR "the aged"[Title/Abstract]) AND ("balance"[Title/Abstract] OR "postural control"[Title/Abstract] OR "falls"[Title/Abstract] OR "Berg Balance Scale"[Title/Abstract] OR "fall risk"[Title/Abstract] OR "gait stability"[Title/Abstract] OR "gait stabilit*"[Title/Abstract] OR "postural stability"[Title/Abstract] OR "postural stabilit*"[Title/Abstract] OR "equilibrium"[Title/Abstract] OR "stability during locomotion"[Title/Abstract]) | 192 |
| Finally included |  | **10** |

**Cochrane Library**

| **Concept** | **Query** | **Hits** |
| --- | --- | --- |
| #1 | ("sensory reweight*" OR "sensory recalibrat*" OR "multisensory integrat*" OR "multisensory combinat*" OR "sensory fus*" OR "sensory train*" OR "sensory strateg*" OR "sensory process*" OR "sensory adapt*") AND ("age*" OR "elderly*" OR "older adult*" OR "senior*" OR "geriatr*" OR "aged population" OR "the aged") AND ("balance" OR "postural control" OR "falls" OR "Berg Balance Scale" OR "fall risk" OR "gait stabilit*" OR "postural stabilit*" OR "equilibrium" OR "stability during locomotion") | 62 |
| Finally included |  | **0** |

**EMBASE**

| **Concept** | **Query** | **Hits** |
| --- | --- | --- |
| #1 | ("sensory reweight*" OR "sensory recalibrat*" OR "multisensory integrat*" OR "multisensory combinat*" OR "sensory fus*" OR "sensory train*" OR "sensory strateg*" OR "sensory process*" OR "sensory adapt*") AND ("age*" OR "elderly*" OR "older adult*" OR "senior*" OR "geriatr*" OR "aged population" OR "the aged") AND ("balance" OR "postural control" OR "falls" OR "Berg Balance Scale" OR "fall risk" OR "gait stabilit*" OR "postural stabilit*" OR "equilibrium" OR "stability during locomotion") | 372 |
| Finally included |  | **0** |

**Web of Science**

| **Concept** | **Query** | **Hits** |
| --- | --- | --- |
| #1 | ("sensory reweight*" OR "sensory recalibrat*" OR "multisensory integrat*" OR "multisensory combinat*" OR "sensory fus*" OR "sensory train*" OR "sensory strateg*" OR "sensory process*" OR "sensory adapt*") AND ("age*" OR "elderly*" OR "older adult*" OR "senior*" OR "geriatr*" OR "aged population" OR "the aged") AND ("balance" OR "postural control" OR "falls" OR "Berg Balance Scale" OR "fall risk" OR "gait stabilit*" OR "postural stabilit*" OR "equilibrium" OR "stability during locomotion") | 349 |
| Finally included |  | **2** |

**Scopus**

| **Concept** | **Query** | **Hits** |
| --- | --- | --- |
| #1 | ("sensory reweight*" OR "sensory recalibrat*" OR "multisensory integrat*" OR "multisensory combinat*" OR "sensory fus*" OR "sensory train*" OR "sensory strateg*" OR "sensory process*" OR "sensory adapt*") AND ("age*" OR "elderly*" OR "older adult*" OR "senior*" OR "geriatr*" OR "aged population" OR "the aged") AND ("balance" OR "postural control" OR "falls" OR "Berg Balance Scale" OR "fall risk" OR "gait stabilit*" OR "postural stabilit*" OR "equilibrium" OR "stability during locomotion") | 346 |
| Finally included |  | **2** |

**Appendix B. Characteristics of included studies**

| **Study** | **Country** | **Sample size** | **Age, mean (SD), years** | **Sensory modality** | **Intervention type** | **Duration and frequency** | **Outcome** | **Main findings** |
| --- | --- | --- | --- | --- | --- | --- | --- | --- |
| Merriman et al [40], 2015 | Ireland | Intervention: 38; control: 38 | Intervention: 74.06 (8.97); control: 74.33 (11.09) | Visual, vestibular, and somatosensory | Dynamic | 5 weeks; 2 days per week; 30 minutes per session | BBS | Balance training was associated with improved BBS, ABC, and FAB scores in the intervention group, with little change in the control group. |
| Bao et al [35], 2018 | United States | Intervention: 6; control: 6 | Intervention: 74.06 (8.97); control: 74.33 (11.09) | Visual, vestibular, and somatosensory | Static | 8 weeks; 3 days per week; 45 minutes per session | AP displacement, ML displacement, and TUG | Both groups showed improvements in AP and ML displacement. The intervention group showed greater improvement over time, whereas TUG time showed no clear between-group difference. |
| Chen et al [11], 2021 | China | Intervention: 16; control: 16 | Intervention: 64.7 (3.0); control: 66.3 (2.7) | Visual, vestibular, and somatosensory | Dynamic | 1 week; 3 days per week; 45 minutes per session | AP displacement and ML displacement | AP displacement decreased after training, indicating reduced postural sway. ML displacement showed little change. |
| Brahms et al [42], 2021 | Germany | Intervention: 10; control: 11 | Intervention: 73.1 (4.8); control: 71.1 (6.2) | Visual and somatosensory | Static | 6 weeks; 3 days per week; 30 minutes per session | TUG | The training intervention showed limited effects on TUG performance and postural control indicators. |
| Sedighi Darijani et al [43], 2024 | Iran | Intervention: 15; control: 20 | Intervention: 65.15 (3.64); control: 65.30 (3.62) | Visual, vestibular, and somatosensory | Dynamic and static | 5 weeks; frequency not reported | TUG | Both groups showed reduced TUG time after the intervention, with similar improvements between groups. |
| Hashim et al [36], 2024 | Canada | Intervention: 5; control: 14 | Intervention: 71.40 (5.00); control: 72.60 (7.80) | Visual, vestibular, and somatosensory | Dynamic and static | 6 weeks; 3 days per week; 30 minutes per session | AP displacement and ML displacement | AP displacement decreased in the intervention group, suggesting improved anterior-posterior stability. ML displacement showed limited change. |
| Tanaka et al [38], 2025 | Japan | Intervention: 30; control: 8 | Intervention: 66.40 (3.60); control: 71.00 (1.90) | Visual, vestibular, and somatosensory | Dynamic and static | 10 weeks; 3 days per week; 15 minutes per session | AP displacement and ML displacement | Trunk and sole stimulation training improved anterior-posterior balance outcomes. ML displacement showed little between-group difference. |
| Zahedian-Nasab et al [41], 2021 | Iran | Intervention: 63; control: 30 | Intervention: 72.00 (7.81); control: 69.67 (7.73) | Visual, vestibular, and somatosensory | Dynamic | 6 weeks; 2 days per week; 60 minutes per session | TUG and BBS | Virtual reality exercise reduced TUG time and improved BBS scores compared with control. |
| Zhou et al [44], 2025 | China | Intervention: 146; control: 61 | Intervention: 84.60 (3.30); control: 84.10 (3.10) | Visual, vestibular, and somatosensory | Dynamic | 12 weeks; 2 days per week; 60 minutes per session | TUG | TUG time showed little change in the intervention group but increased in the control group. |
| Mirelman et al [47], 2016 | Ireland | Intervention: 14; control: 136 | Intervention: 73.3 (6.40); control: 74.2 (6.90) | Visual, vestibular, and somatosensory | Dynamic | 6 weeks; 3 days per week; 45 minutes per session | Fall risk | Fall risk decreased in the intervention group, and gait variability during obstacle negotiation was lower. |
| Chen et al [39], 2020 | China | Intervention: 24; control: 14 | Intervention: 72.2 (2.8); control: 75.1 (5.5) | Visual, vestibular, and somatosensory | Dynamic | 8 weeks; 3 days per week; 30 minutes per session | TUG and BBS | Augmented reality-assisted Tai Chi training reduced TUG time and improved BBS scores. |
| Rezaei et al [46], 2021 | Iran | Intervention: 76; control: 24 | Intervention: 70.75 (4.68); control: 69.95 (4.53) | Visual, vestibular, and somatosensory | Dynamic and static | 4 weeks; 3 days per week; 30 minutes per session | Fall risk | Both neurofeedback and balance exercise reduced fall risk indices and improved balance-related outcomes. |
| Gianoudis et al [45], 2014 | Australia | Intervention: 13; control: 81 | Intervention: 67.2 (5.5); control: 67.7 (6.5) | Visual, vestibular, and somatosensory | Dynamic | 12 weeks; 3 days per week; 90 minutes per session | TUG and fall risk | The targeted multimodal exercise program showed limited differences between groups in TUG performance. |
| Shahrbanian et al [37], 2021 | Iran | Intervention: 13; control: 13 | Intervention: 68.03 (4.96); control: 66.4 (9.3) | Visual, vestibular, and somatosensory | Static | 12 weeks; 3 days per week; 30 minutes per session | AP displacement and fall risk | Physical activity and neurofeedback training improved AP displacement and reduced fall risk compared with control. |

Abbreviations: AP, anterior-posterior; BBS, Berg Balance Scale; ML, medio-lateral; TUG, Timed Up and Go.

**Appendix C. Forest plot summary**

**
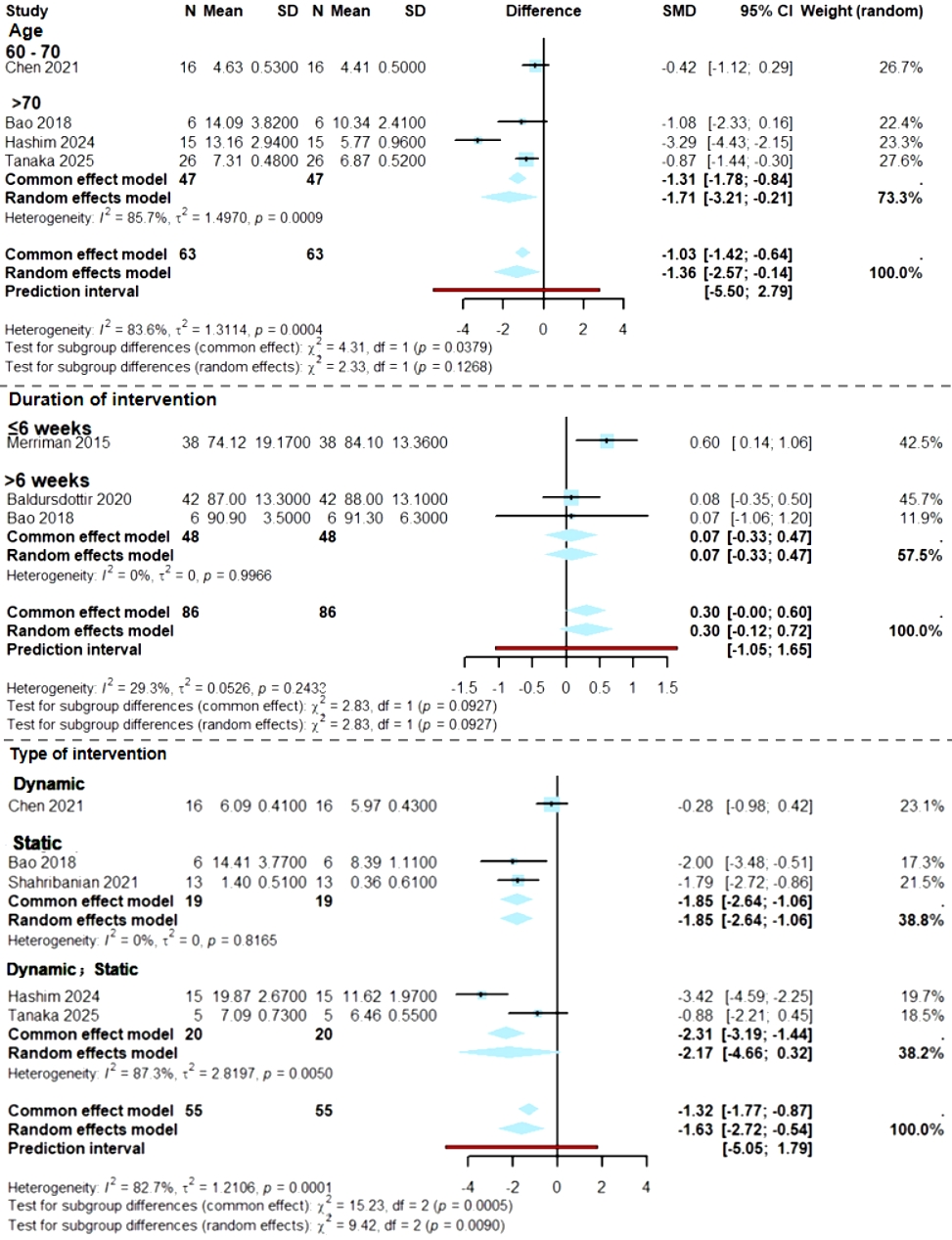
**

**Figure C1. Group the studies by age, intervention duration, and intervention type to analyze the improvement effects of the AP displacement under different conditions.**

**
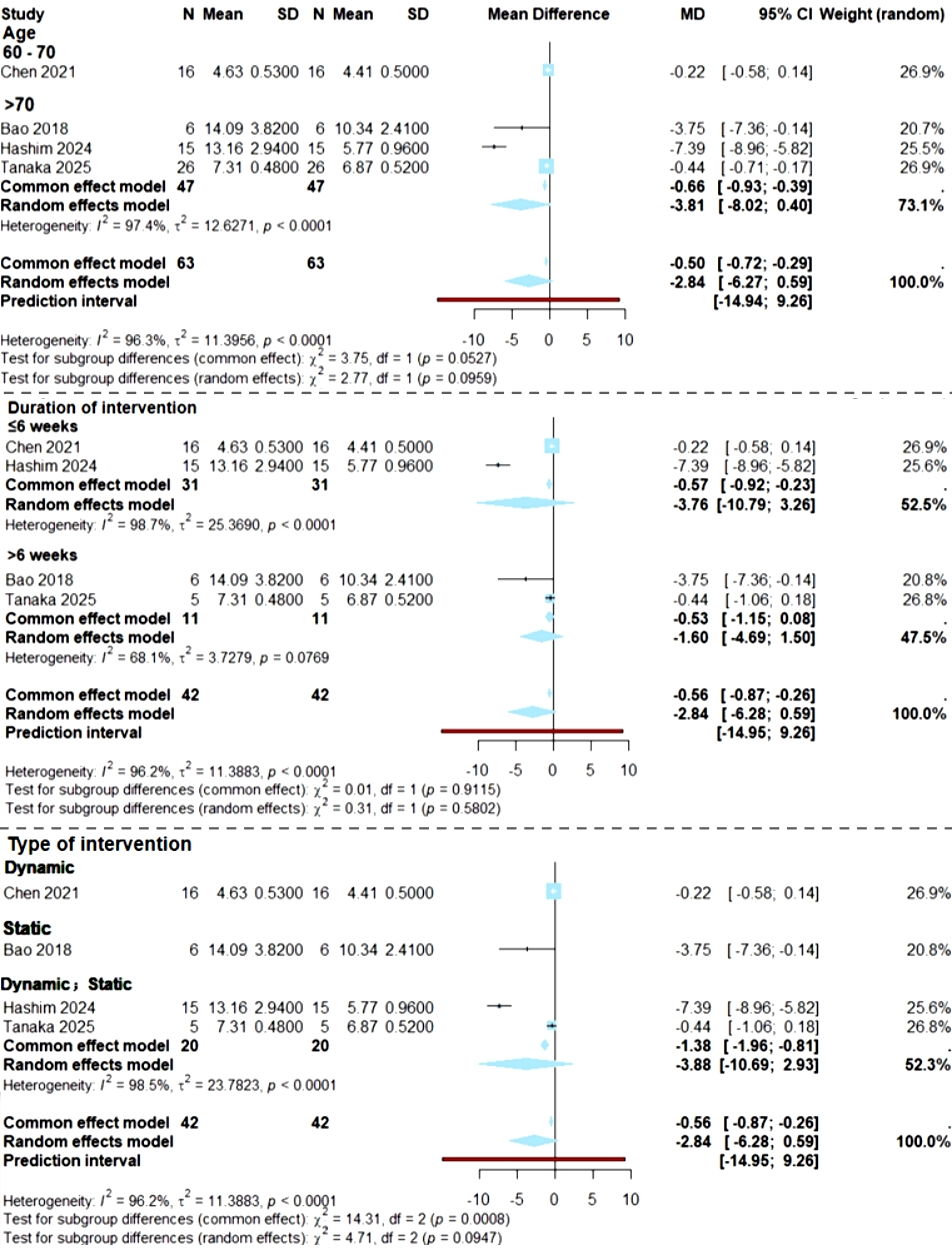
**

**Figure C2. Group the studies by age, intervention duration, and intervention type to analyze the improvement effects of the ML displacement under different conditions.**

**
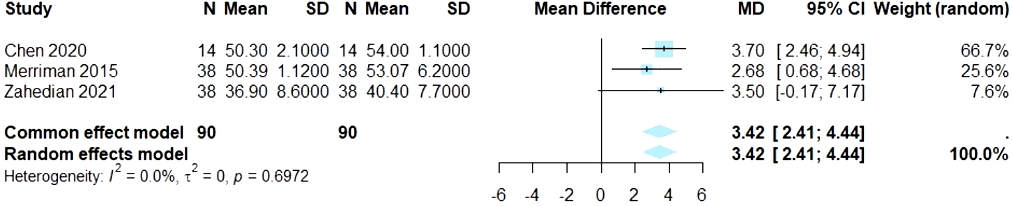
**

**Figure C3. Forest plot for mean difference in BBS scores before and after intervention.**

**
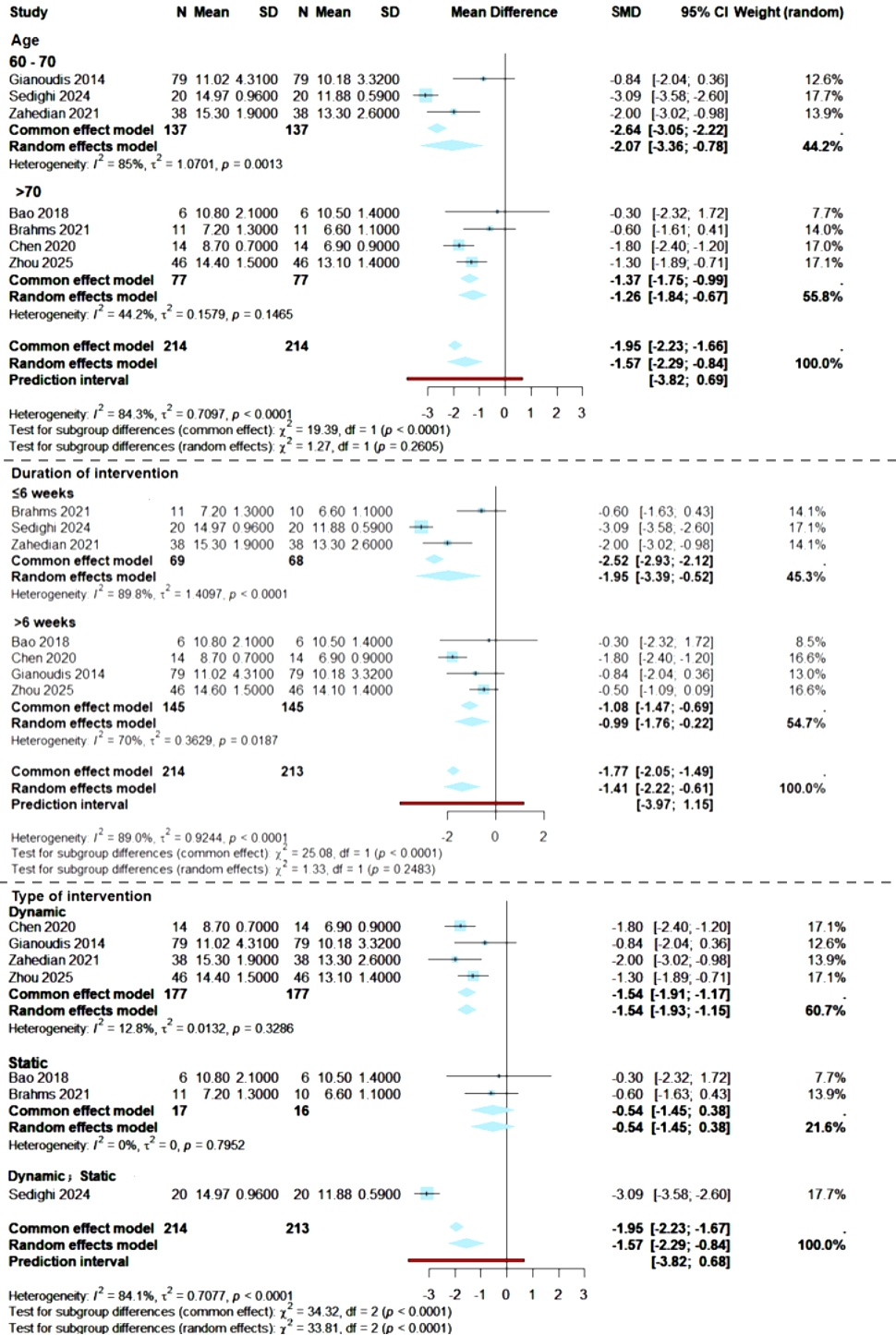
**

**Figure C4. Group the studies according to age, duration of intervention, and type of intervention to analyze the improvement effects of the Timed Up and Go (TUG) test under different conditions.**

**
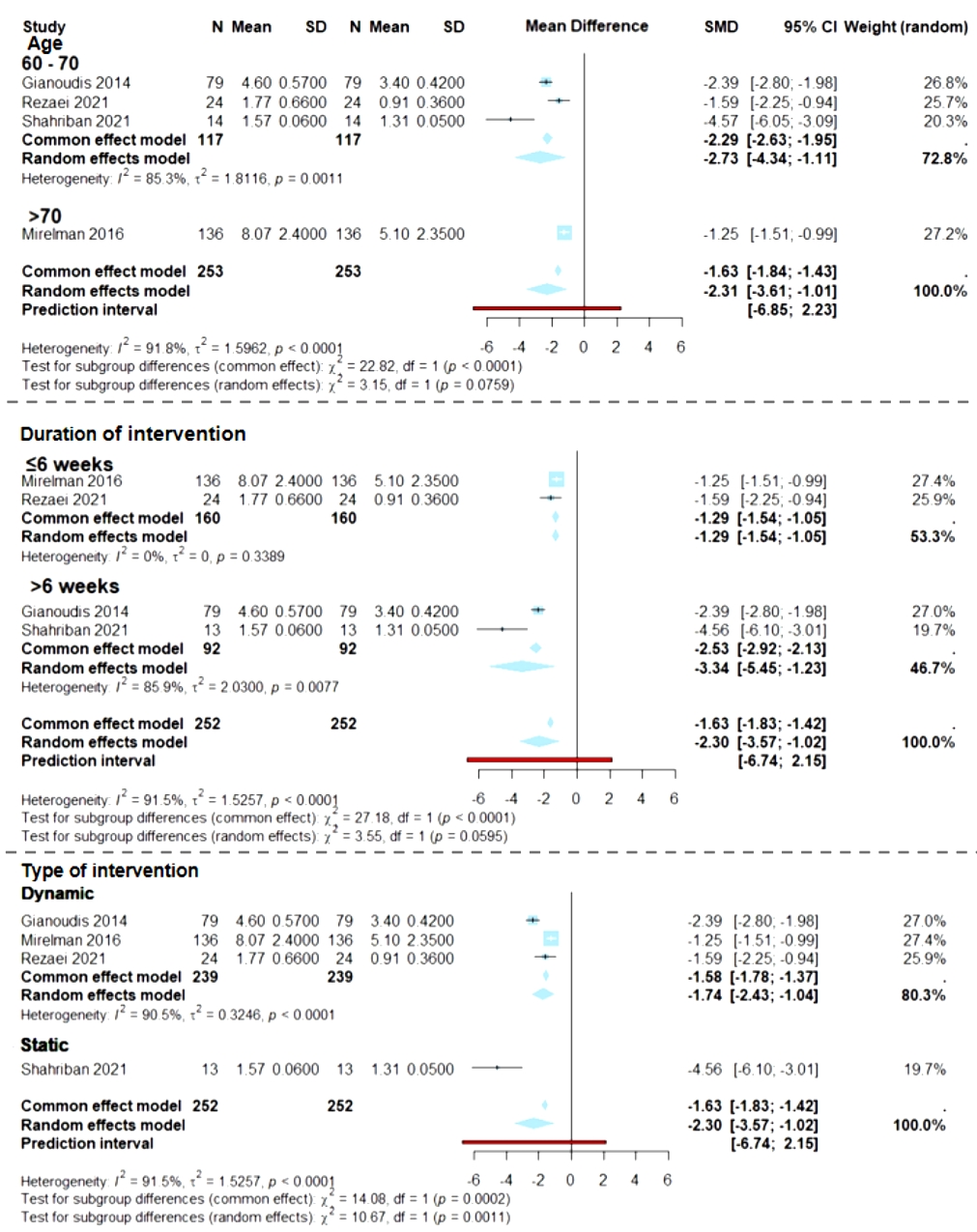
**

**Figure C5. Grouped analysis of fall risk by age, intervention duration, and intervention type.**

**Appendix D. Meta-regression**

***Changes in heterogeneity***

Below we present the results from the changes in heterogeneity in each meta-regression model.

| Covariate | β (95% CI) | Standard error | *P* value |
| --- | --- | --- | --- |
| **Center of Pressure displacement in the anterior-posterior** | | | |
| Average age | −0.257 (−0.290 to 0.217) | 4.18 | .68 |
| Duration of intervention | −0.561 (−0.154 to 0.334) | 4.03 | .33 |
| Type of intervention | −0.581 (−0.079 to 0.035) | 0.94 | .31 |
| **Center of Pressure displacement in the medio-lateral** | | | |
| Average age | 0.126 (−0.384 to 0.418) | 5.01 | .87 |
| Duration of intervention | 0.696 (−0.185 to 0.359) | 3.40 | .30 |
| Type of intervention | 0.469 (−0.068 to 0.097) | 1.04 | .53 |

Abbreviation: CI, confidence interval.

**Appendix E. Funnel plot**


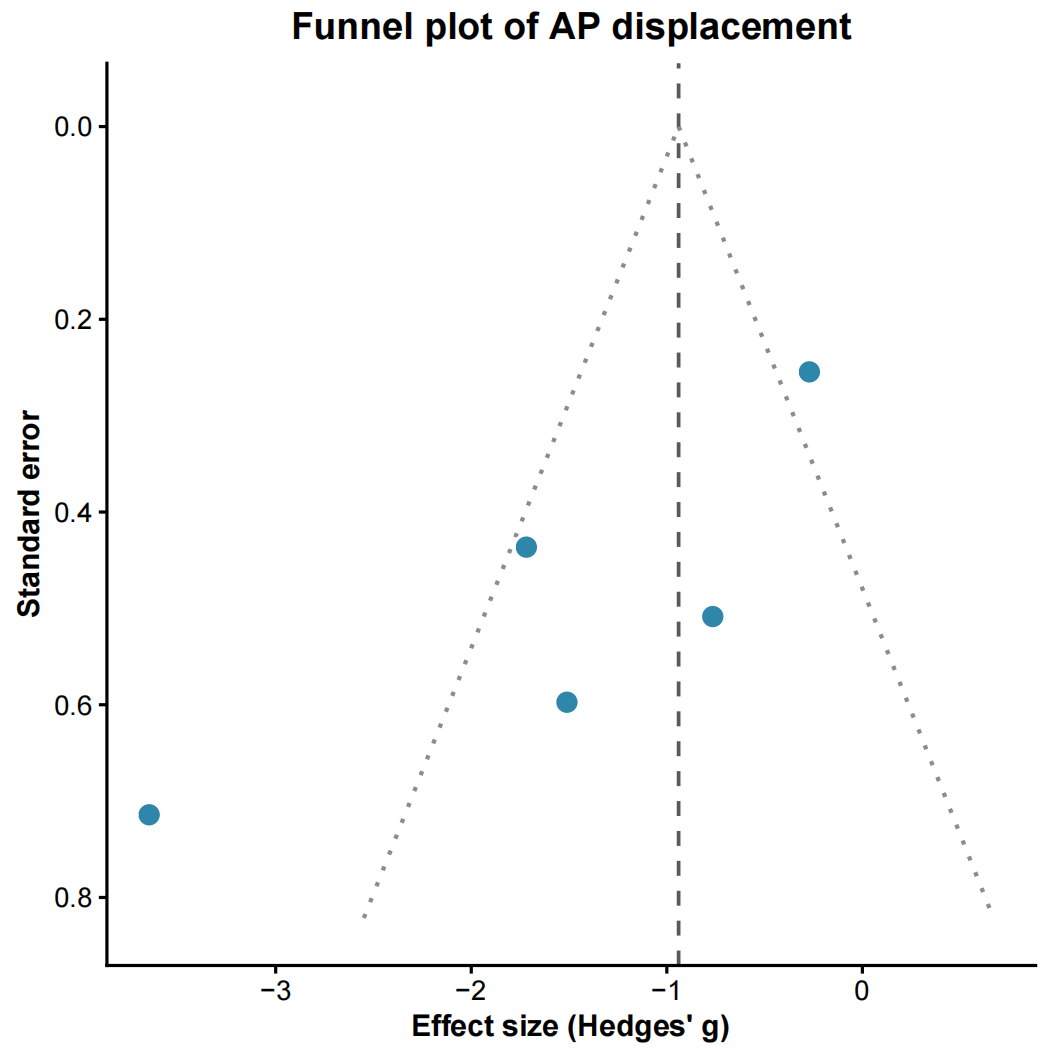


**Figure E1. Funnel plot of standardized mean differences in anterior-posterior displacement.**


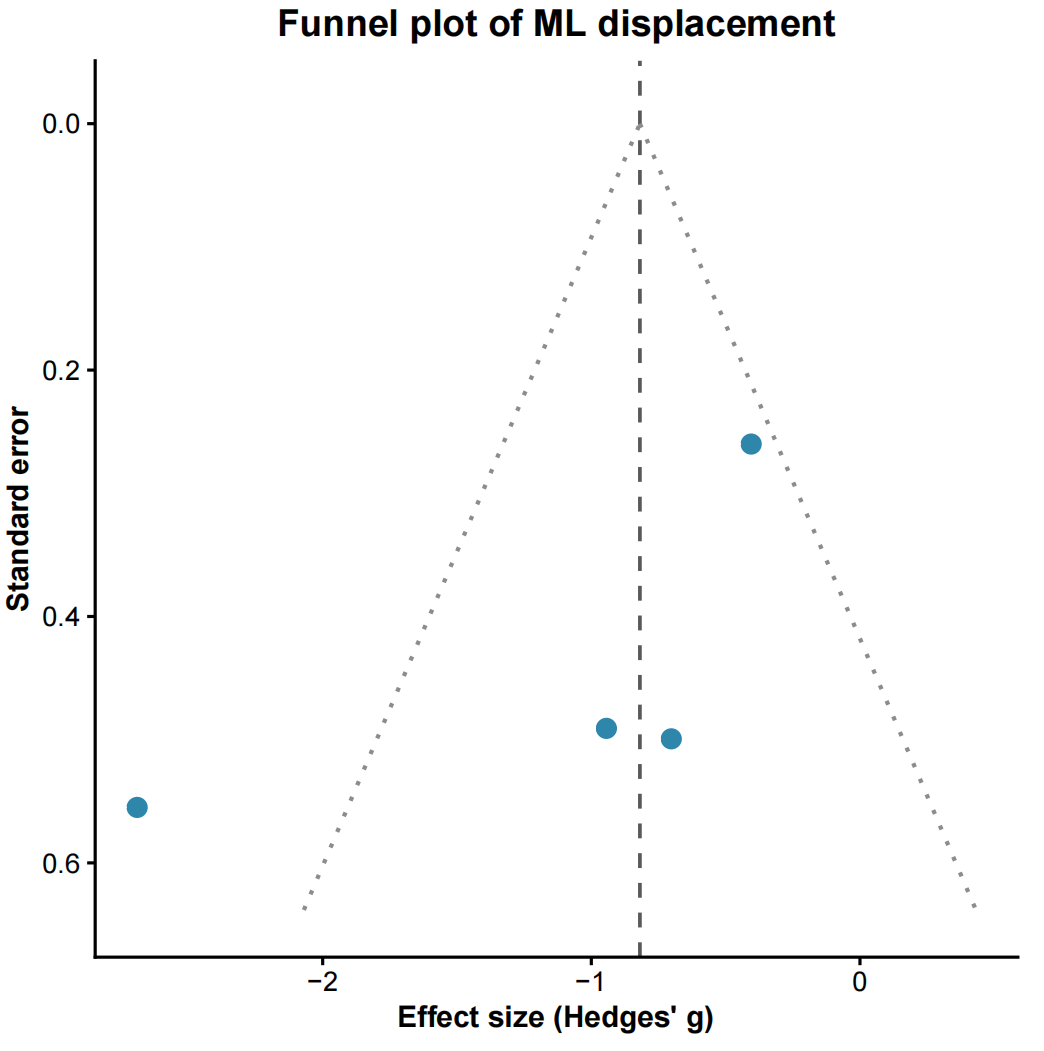


**Figure E2. Funnel plot of standardized mean differences in medio-lateral displacement.**


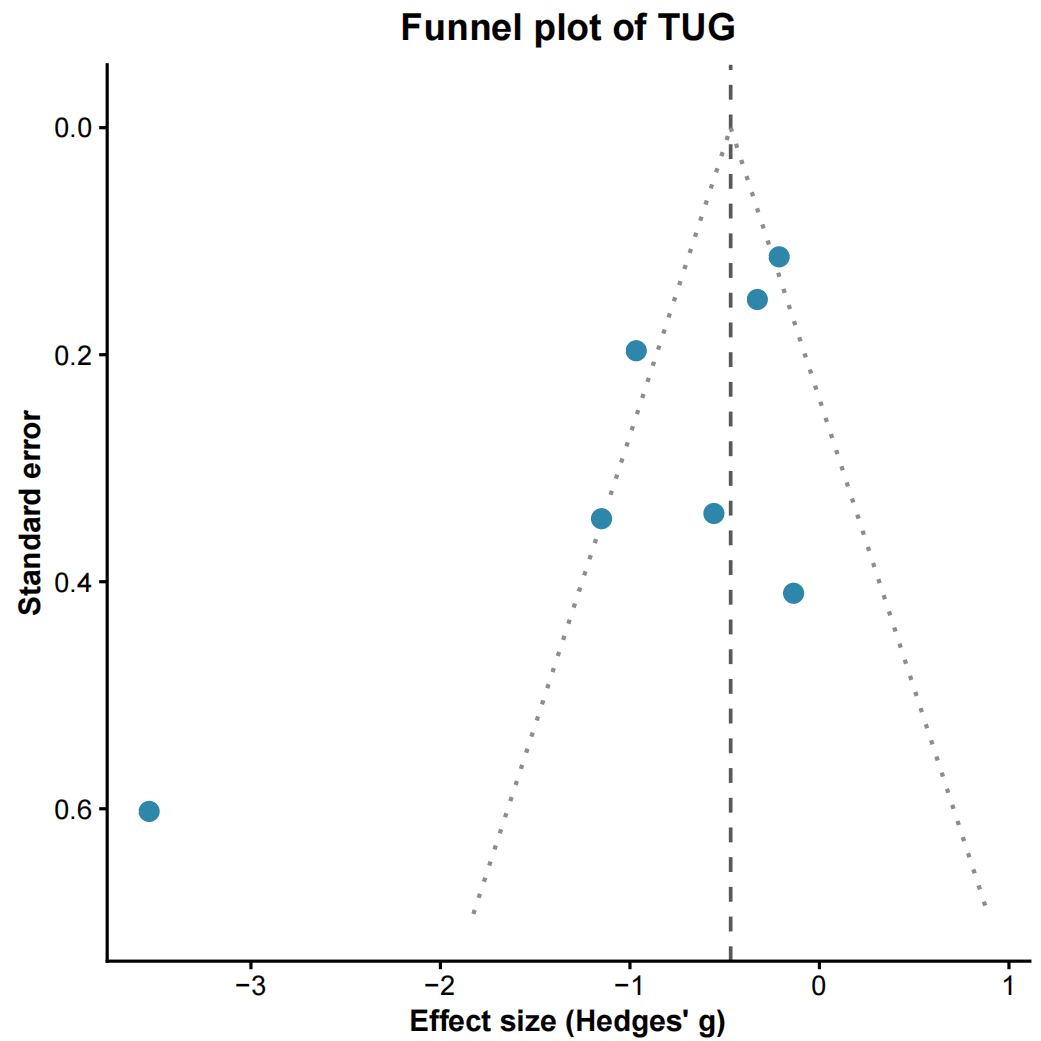


**Figure E3. Funnel plot of standardized mean differences for timed up and go test.**


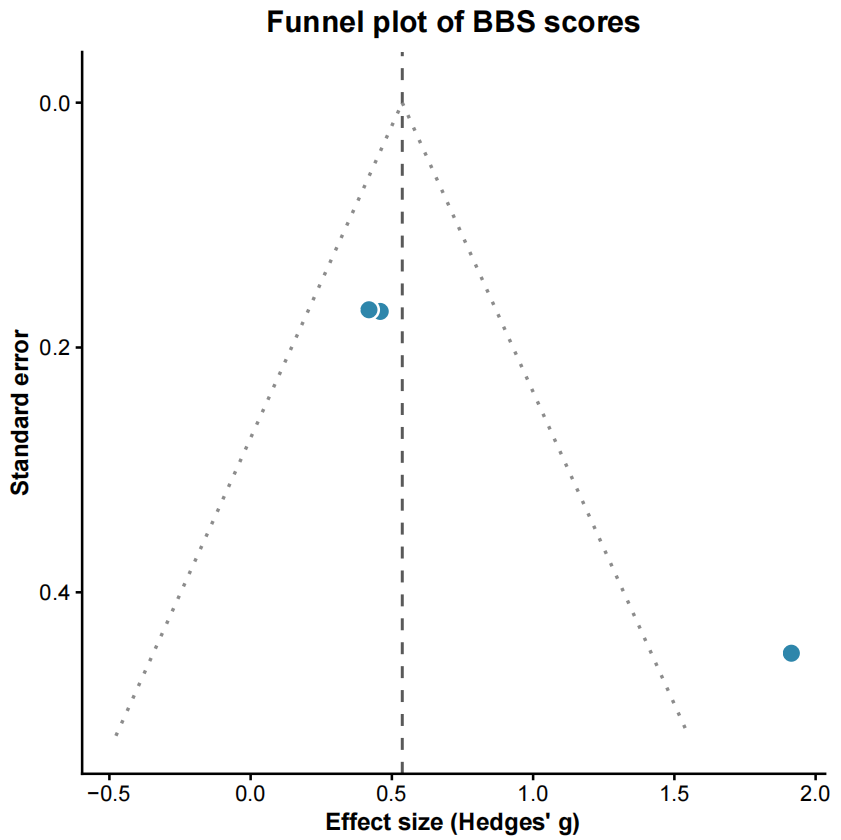


**Figure E4. Funnel plot of mean differences for Berg Balance Scale scores.**


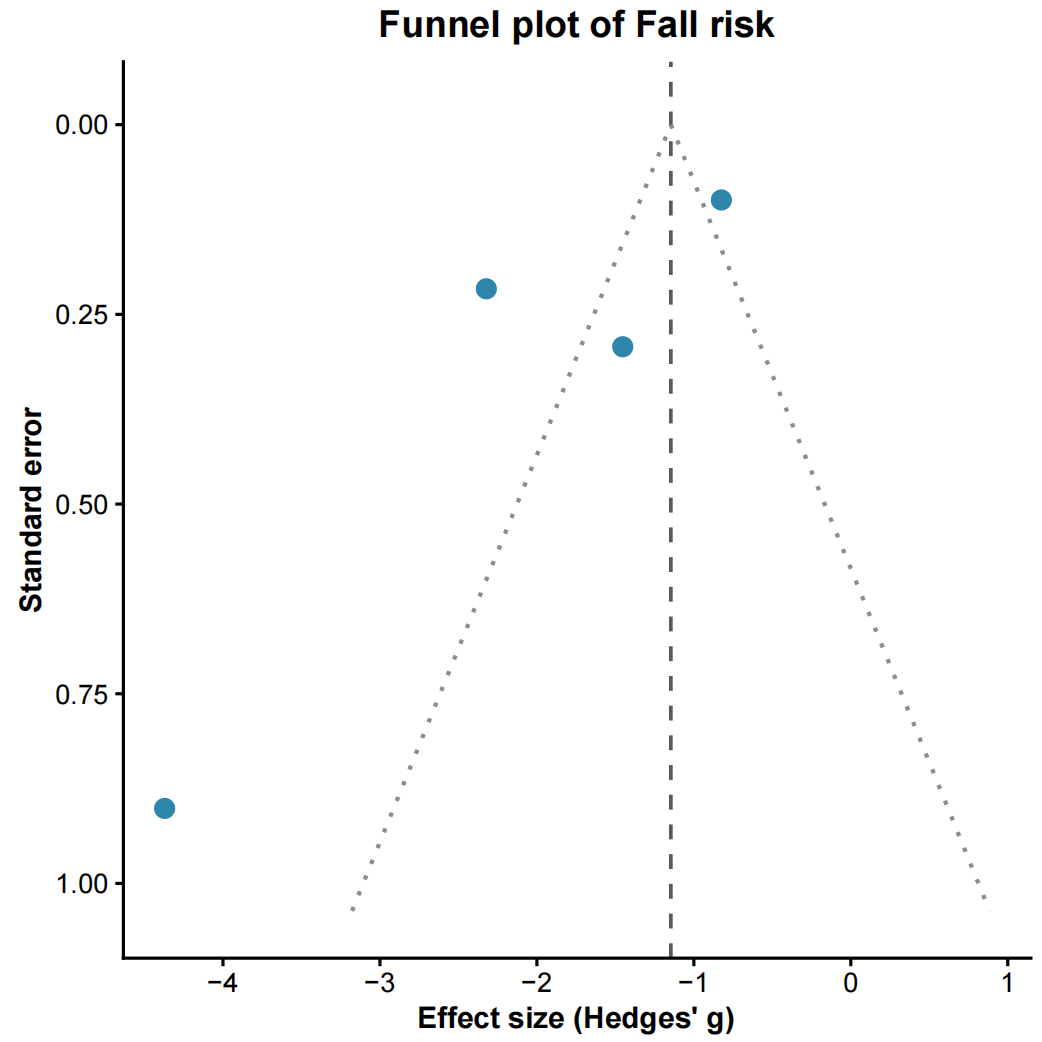


**Figure E5. Funnel plot of standardized mean differences for fall risk.**

**Appendix F. Research dataset**

| **Indicator** | **Study** | **Before** | | | **After** | | |
| --- | --- | --- | --- | --- | --- | --- | --- |
|  |  | **Mean** | **SD** | **Total** | **Mean** | **SD** | **Total** |
| AP displacement | Bao et al [35], 2018 | 14.41 | 3.77 | 6 | 8.39 | 1.11 | 6 |
|  | Chen et al [11], 2021 | 6.09 | 0.41 | 16 | 5.97 | 0.43 | 16 |
|  | Hashim et al [36], 2024 | 19.87 | 2.67 | 15 | 10.62 | 1.97 | 15 |
|  | Shahrbanian et al [37], 2021 | 1.4 | 0.51 | 13 | 0.36 | 0.61 | 13 |
|  | Tanaka et al [38], 2025 | 7.09 | 0.73 | 5 | 6.46 | 0.55 | 5 |
| ML displacement | Bao et al [35], 2018 | 14.09 | 3.82 | 6 | 10.34 | 2.41 | 6 |
|  | Chen et al [11], 2021 | 4.63 | 0.53 | 16 | 4.41 | 0.5 | 16 |
|  | Hashim et al [36], 2024 | 13.16 | 2.94 | 15 | 5.77 | 0.96 | 15 |
|  | Tanaka et al [38], 2025 | 7.31 | 0.48 | 5 | 6.87 | 0.52 | 5 |
| BBS Scores | Chen et al [39], 2020 | 50.3 | 2.1 | 14 | 54 | 1.1 | 14 |
|  | Merriman et al [40], 2015 | 50.39 | 1.12 | 38 | 53.07 | 6.2 | 38 |
|  | Zahedian-Nasab et al [41], 2021 | 36.9 | 8.6 | 38 | 40.4 | 7.7 | 38 |
| TUG | Bao et al [35], 2018 | 10.8 | 2.1 | 6 | 10.5 | 1.4 | 6 |
|  | Brahms et al [42], 2021 | 7.2 | 0.3 | 11 | 6.6 | 1.1 | 10 |
|  | Chen et al [39], 2020 | 8.7 | 1.7 | 14 | 6.9 | 0.9 | 14 |
|  | Gianoudis et al [45], 2014 | 11.02 | 4.31 | 79 | 10.18 | 3.32 | 79 |
|  | Sedighi Darijani et al [43], 2024 | 14.97 | 0.96 | 20 | 11.88 | 0.59 | 20 |
|  | Zahedian-Nasab et al [41], 2021 | 15.3 | 1.9 | 38 | 13 | 2.6 | 38 |
|  | Zhou et al [44], 2025 | 14.6 | 1.5 | 46 | 14.1 | 1.5 | 46 |
| Fall Risk | Gianoudis et al [45], 2014 | 4.6 | 0.57 | 79 | 3.4 | 0.42 | 79 |
|  | Mirelman et al [47], 2016 | 8.07 | 2.4 | 136 | 6.1 | 2.35 | 136 |
|  | Rezaei et al [46], 2021 | 1.77 | 0.66 | 24 | 0.91 | 0.36 | 24 |
|  | Shahrbanian et al [37], 2021 | 1.57 | 0.06 | 13 | 1.31 | 0.05 | 13 |

Abbreviations: AP, anterior-posterior; BBS, Berg Balance Scale; ML, medio-lateral; TUG, Timed Up and Go.

**References cited in Multimedia Appendix 1**

11. Chen YC, Chou YC, Hwang IS. Reliance on visual input for balance skill transfer in older adults: EEG connectome analysis using minimal spanning tree. Front Aging Neurosci. 2021;13:632553. doi: 10.3389/fnagi.2021.632553.

35. Bao T, Carender WJ, Kinnaird C, et al. Effects of long-term balance training with vibrotactile sensory augmentation among community-dwelling healthy older adults: a randomized preliminary study. J Neuroeng Rehabil. 2018;15(1):5. doi: 10.1186/s12984-017-0339-6.

36. Hashim J, Campelo AM, Doyle-Baker PK, Cossich VR, Katz L. Comparison between six-week exergaming, conventional balance and no exercise training program on older adults’ balance and gait speed. Int J Comput Sci Sport. 2024;23(1):93-109. doi: 10.2478/ijcss-2024-0006.

37. Shahrbanian S, Hashemi A, Hemayattalab R. The comparison of the effects of physical activity and neurofeedback training on postural stability and risk of fall in elderly women: a single-blind randomized controlled trial. Physiother Theory Pract. 2021;37(2):271-278. doi: 10.1080/09593985.2019.1630877.

38. Tanaka T, Maeda Y, Miura T. Effects of tactile sensory stimulation training of the trunk and sole on standing balance ability in older adults: a randomized controlled trial. J Funct Morphol Kinesiol. 2025;10(1):96. doi: 10.3390/jfmk10010096.

39. Chen PJ, Penn IW, Wei SH, Chuang LR, Sung WH. Augmented reality-assisted training with selected Tai-Chi movements improves balance control and increases lower limb muscle strength in older adults: a prospective randomized trial. J Exerc Sci Fit. 2020;18(3):142-147. doi: 10.1016/j.jesf.2020.05.003.

40. Merriman NA, Whyatt C, Setti A, Craig C, Newell FN. Successful balance training is associated with improved multisensory function in fall-prone older adults. Comput Hum Behav. 2015;45:192-203. doi: 10.1016/j.chb.2014.12.017.

41. Zahedian-Nasab N, Jaberi A, Shirazi F, Kavousipor S. Effect of virtual reality exercises on balance and fall in elderly people with fall risk: a randomized controlled trial. BMC Geriatr. 2021;21(1):509. doi: 10.1186/s12877-021-02462-w.

42. Brahms M, Heinzel S, Rapp M, et al. Cognitive-postural multitasking training in older adults - effects of input-output modality mappings on cognitive performance and postural control. J Cogn. 2021;4(1):20. doi: 10.5334/joc.146.

43. Sedighi Darijani S, Sahebozamani M, Eslami M, Babakhanian S, Alimoradi M, Iranmanesh M. The effect of neurofeedback and somatosensory exercises on balance and physical performance of older adults: a parallel single-blinded randomized controlled trial. Sci Rep. 2024;14(1):24087. doi: 10.1038/s41598-024-74980-7.

44. Zhou J, Liu B, Xu JF, et al. Home-based strength and balance exercises for fall prevention among older individuals of advanced age: a randomized controlled single-blind study. Ann Med. 2025;57(1):2459818. doi: 10.1080/07853890.2025.2459818.

45. Gianoudis J, Bailey CA, Ebeling PR, et al. Effects of a targeted multimodal exercise program incorporating high-speed power training on falls and fracture risk factors in older adults: a community-based randomized controlled trial. J Bone Miner Res. 2014;29(1):182-191. doi: 10.1002/jbmr.2014.

46. Rezaei K, Nami M, Sinaei E, Bagheri Z, Kordi Yoosefinejad A. A comparison between effects of neurofeedback and balance exercise on balance of healthy older adults. J Biomed Phys Eng. 2021;11(6):713-722. doi: 10.31661/jbpe.v0i0.1203.

47. Mirelman A, Rochester L, Maidan I, et al. Addition of a non-immersive virtual reality component to treadmill training to reduce fall risk in older adults (V-TIME): a randomised controlled trial. Lancet. 2016;388(10050):1170-1182. doi: 10.1016/S0140-6736(16)31325-3.
